# Association Between Non-Dipping and Fragmented QRS Complexes in Prehypertensive Patients

**DOI:** 10.5935/abc.20180242

**Published:** 2019-01

**Authors:** Mehmet Eyuboglu, Bahri Akdeniz

**Affiliations:** 1 Department of Cardiology - Medical Park Izmir Hospital, Karsiyaka, Izmir - Turkey; 2 Department of Cardiology - Dokuz Eylul University Hospital, Izmir - Turkey

**Keywords:** Prehypertension, Hypertension, Electrocardiography, Fragmented QRS, Ambulatory Blood Pressure Monitoring, Non-dipping

## Abstract

**Background:**

Fragmented QRS (fQRS) is a sign of adverse cardiovascular events in various cardiovascular diseases. It is also associated with increased blood pressure and non-dipping in hypertensive patients. However, no study has investigated the importance of fQRS in prehypertensive patients.

**Objectives:**

The aim of our study is to investigate the relationship between fQRS and non-dipper status in prehypertensive patients.

**Methods:**

Two hundred and sixteen eligible, newly diagnosed prehypertensive patients who underwent 24-hour ambulatory blood pressure monitoring (ABPM) for further evaluation of blood pressure between June 2015 and July 2016 were included into the study. Patients were divided into three groups according to ABPM results: normotensives, dipper prehypertensives, and non-dipper prehypertensives. Groups were compared regarding presence of fQRS on electrocardiography. Additionally, multinomial logistic regression analysis was used to determine the relationship between fQRS and blood pressure pattern in prehypertensive patients.

**Results:**

According to ABPM recordings, 61 patients had normotensive blood pressure pattern (systolic blood pressure < 120 mmHg and diastolic blood pressure < 80 mmHg). Of the remaining 155 prehypertensive patients, 83 were dippers and 72 were non-dippers. Non-dipper prehypertensives had a significantly higher frequency of fQRS compared to normotensives (p = 0.048). Furthermore, multinomial logistic regression analysis revealed that fQRS is an independent predictor of non-dipping blood pressure pattern in prehypertensive patients (p = 0.017, OR: 4.071, 95% CI: 1.281-12.936).

**Conclusions:**

We found that fQRS is a predictor of non-dipping in prehypertensives. As a marker of fibrosis and higher fibrotic burden within myocardium, fQRS may be useful in identifying high-risk prehypertensive patients before the development of hypertension.

## Introduction

Increased blood pressure is one of the leading causes of cardiovascular morbidity and mortality around the globe. Because of the difficulties involved in diagnosing prehypertension, the definition of prehypertension remains controversial. Prehypertension is not a benign condition; it indicates future hypertension and adverse cardiovascular events and is generally defined as systolic blood pressure (SBP) of 120-139 mmHg and/or diastolic blood pressure (DBP) of 80-89 mmHg.^1^^,^^2^ Normal blood pressure has a circadian variability with a morning surge and reduction during the rest of the day with a 10% to 20% decline at nighttime, and this phenomenon is known as dipping. Non-dipping pattern, which is defined as less than 10% decrease in blood pressure levels at nighttime, is associated with worse adverse cardiovascular events compared to dipping blood pressure pattern.^3^^,^^4^

A narrow fragmented QRS complex (fQRS) on electrocardiography (ECG) is a sign of inhomogeneous and delayed ventricular conduction and is associated with myocardial scarring, fibrosis, and adverse cardiovascular events in various cardiovascular diseases.^5^^-^^7^ It is defined by the presence of notches in the R or S wave in two contiguous leads in one of the major coronary artery territories without a typical bundle branch block and with a QRS duration of < 120 milliseconds.^8^ Importantly, increased blood pressure is associated with presence of fQRS on ECG.^9^ Furthermore, non-dipper hypertensive patients have higher frequency of fQRS on ECG compared to dippers, thus indicating myocardial fibrosis and higher fibrotic burden in non-dippers.^10^^,^^11^ However, the importance and usefulness of fQRS in prehypertensive patients is not clear. The present study aimed to investigate the relationship between prehypertensive blood pressure patterns and the presence of fQRS on ECG to identify the myocardial fibrotic burden and risk assessment of prehypertensive subjects before the development of hypertension.

## Methods

### Patient selection

**Figure 1:**
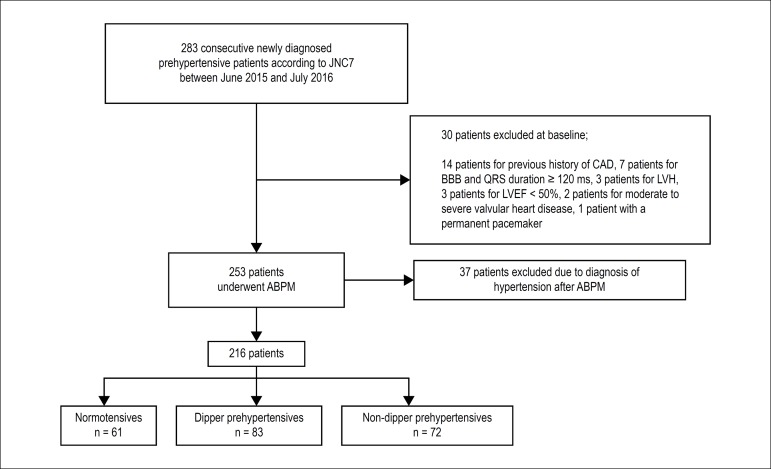
Flow chart of the study design. JNC7: Seventh Report of the Joint National Committee on Prevention, Detection, Evaluation, and Treatment of High Blood Pressure, CAD: coronary artery disease; BBB: bundle branch block; LVH: left ventricular hypertrophy; LVEF: left ventricular ejection fraction; ABPM: 24-hour ambulatory blood pressure monitoring.

A total of 283 consecutive patients who were defined as newly diagnosed prehypertensive patients after routine cardiac examination at our outpatient clinic between June 2015 and July 2016 were screened for the study. Prehypertension was defined as SBP of 120-139 mmHg and/or a DBP of 80-89 mmHg in accordance with the Seventh Report of the Joint National Committee on Prevention, Detection, Evaluation, and Treatment of High Blood Pressure (JNC7).^1^ Figure 1 demonstrates the flow chart of our study design. Subsequently, all patients underwent 24-hour ambulatory blood pressure monitoring (ABPM) for final blood pressure pattern diagnosis. Of the patients screened, 67 were excluded from the study: 37 who were diagnosed with hypertension after 24-hour ABPM recordings, fourteen with a history of coronary artery disease (CAD), seven with complete or incomplete bundle branch block and QRS duration ≥ 120 ms, three with left ventricular hypertrophy (LVH), three with left ventricular ejection fraction (LVEF) < 50%, two with moderate to severe valvular heart disease, and one with a permanent pacemaker. Consequently, 216 patients were included into the study. Data regarding patients’ medical history were recorded on admission. All biochemical analyses were conducted after an overnight fast. Hypertension was defined as 24-hour mean SBP of ≥ 130 mmhg and/or DBP ≥ 80 mmhg and/or daytime mean SBP ≥ 135 mmhg and/or DBP ≥ 85 mmhg on ABPM recordings.^4^^,^^12^ Diabetes mellitus was defined as at least two fasting plasma glucose levels of ≥ 126 mg/dL, two-hour plasma glucose levels of ≥200 mg/dL, or treatment with antidiabetic drugs, and smoking was defined as the regular use of cigarettes. All patients underwent a detailed echocardiographic examination, and LVH was defined based on electrocardiographic modified Sokolow-Lyon index and/or an increased left ventricular mass index of > 95 g/m^2^ for women and >115 g/m^2^ for men, detected by echocardiography.^12^

The study protocol complied with the Declaration of Helsinki and was approved by the local ethics committee.

### 24-h ABPM recordings

Final diagnoses of blood pressure level and pattern were made based on ABPM recordings. All measurements were taken with an oscillometric device. The cuff was placed on the non-dominant arm and automated recordings were obtained every 30 minutes during 24-hours. Recordings were made on working days and patients were encouraged to undertake their normal daily activities. If >20% of the ABPM recordings were invalid, the test was repeated. Sleep durations were evaluated based on the information obtained from the patients, and no patient reported a change in the daily sleeping and waking periods linked to the ABPM device. The 24-h mean and the daytime and nighttime blood pressure values were calculated for each patient from ABPM recordings. Dipper blood pressure pattern was described as more than 10% decline in SBP and DBP at nighttime and non-dipper pattern was defined as less than 10% decline in SBP and DBP at nighttime.^4^^,^^12^

### Electrocardiography

**Figure 2:**
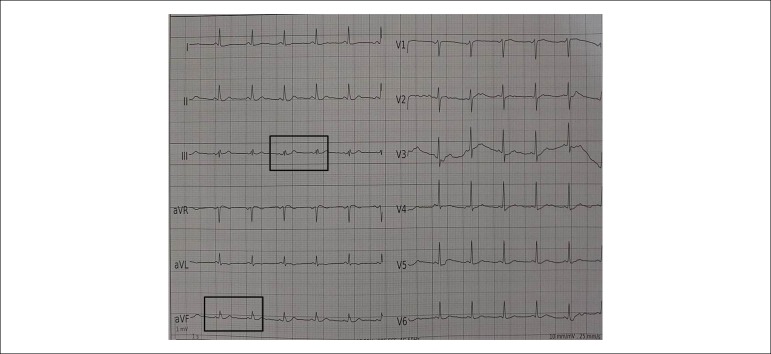
An example of fragmented QRS in our study population.

A standard 12-lead surface ECG was performed on all patients and blindly analyzed by two independent cardiologists. When there was a disagreement, the final decision on the presence of fQRS was reached by consensus. A narrow fQRS complex was defined as the presence of various RSR’ patterns, or notching in R or S waves in the absence of typical bundle branch block in at least two contiguous leads in one of the major coronary artery territories in the original QRS complex^8^ (Figure 2).

### Statistical analysis

Statistical analyses were performed with SPSS (Inc, Chicago, Illinois) version 22.0. Continuous variables were expressed as mean ± standard deviation/median (25-75 percentiles) according to normality and distribution characteristics and were compared using one-way ANOVA, independent samples t-test, or Mann-Whitney U-test, according to group number and distribution characteristics. Categorical variables were expressed as number and percentage (%) and were compared using the χ2 test or the Fisher exact test. Multinomial logistic regression analysis (using normotensive patients as the reference category) was used to determine the relationship between fQRS and blood pressure pattern in prehypertensive patients. Impact significance was reported as odds ratio (OR) and corresponding 95% confidence interval (CI). P < 0.05 was considered significant in all statistical analyses.

## Results

The patients were divided into three groups based on 24-hour ABPM recordings. According to ABPM recordings, 61 patients had a normotensive blood pressure pattern (SBP < 120 mmHg and DBP < 80 mmHg), and we designated these patients as the control group. Of the remaining 155 prehypertensive patients, 83 had dipper blood pressure pattern and 72 had non-dipper pattern. The mean age of the study population was 50.5 years, with 45.8% being female. The frequency of fQRS was 13.9%. The groups were similar regarding cardiovascular risk factors, laboratory parameters, and clinical characteristics. The baseline characteristics, laboratory parameters, and blood pressure levels of the groups are presented in Table 1. Statistical analysis revealed a statistically significant difference between the groups regarding presence of fQRS (p = 0.028). This difference was mainly due to higher frequency of fQRS in non-dipper prehypertensives than in normotensives. Despite the higher frequency of fQRS in non-dippers than in dippers, there was no statistically significant difference regarding the presence of fQRS between non-dipper prehypertensives and dipper prehypertensives (p = 0.400). A similar condition was observed between dipper prehypertensives and the control group (p = 0.784). However, non-dipper prehypertensives had a significantly higher frequency of fQRS than normotensives (p = 0.048). Furthermore, multinomial logistic regression analysis revealed that the presence of fQRS on ECG is an independent predictor of non-dipping blood pressure pattern in prehypertensive patients (p = 0.017, OR: 4.071, 95% CI: 1.281-12.936), (Table 2).

**Table 1:** Baseline demographic and clinical characteristics of the study population according to blood pressure pattern

		All Patients (n:216)		Control (n:61)		Dippers (n:83)		Non-dippers (n:72)		p*
Age (years)		50.5	± 4.3	50.7	± 4.5	50.1	± 4.6	50.7	± 3.7	0.651
Female gender, n (%)		99	(45.8)	30	(49.2)	39	(47.0)	30	(41.7)	0.664
Diabetes, n (%)		18	(8.3)	5	(8.2)	7	(8.4)	6	(8.3)	0.999
Smoking, n (%)		38	(17.6)	10	(16.4)	11	(13.3)	17	(23.6)	0.232
Fragmented QRS, n (%)		30	(13.9)	4	(6.6)	10	(12.0)	16	(22.2)	0.028
Number of leads with fragmented	2	27	(90.0)	4	(100.0)	9	(90.0)	14	(87.5)	0.765
QRS, n (%)	3	3	(10.0)	0	(0.0)	1	(10.0)	2	(12.5)	
24h mean SBP, mmHg		122.5	± 5.2	114.8	± 1.7	124.8	± 2.1	126.4	± 1.7	< 0.001
24h mean DBP, mmHg		74.3	± 5.3	66.2	± 1.8	77.1	± 1.2	78.0	± 1.0	< 0.001
Day SBP, mmHg		128.7	± 1.0	116.2	± 1.6	128.9	± 1.1	128.4	± 0.8	< 0.001
Day DBP, mmHg		78.9	± 1.0	66.0	± 1.8	78.8	± 1.2	79.1	± 0.6	0.175
Night SBP, mmHg		117.6	± 3.4	113.4	± 1.7	114.8	± 2.1	120.8	± 0.8	< 0.001
Night DBP, mmHg		72.0	± 3.4	66.4	± 1.7	69.0	± 0.9	75.5	± 1.5	< 0.001
LVEF (%)		63.1	± 2.4	63.2	± 2.4	62.8	± 2.5	63.3	± 2.4	0.396
Hemoglobin (g/dl)		14.3	± 1.5	14.0	± 1.5	14.5	± 1.5	14.4	± 1.5	0.175
WBC (10^3^/ml)		7.7	± 1.0	7.9	± 0.9	7.5	± 1.1	7.8	± 1.0	0.071
Creatinine (mg/dl)		0.8	± 0.1	0.8	± 0.1	0.8	± 0.1	0.8	± 0.1	0.688
LDL (mg/dl)		108.8	± 19.7	109.5	± 18.3	106.8	± 20.7	110.6	± 19.7	0.359
HDL (mg/dl)		43.0	± 6.2	43.4	± 6.2	43.8	± 6.1	41.7	± 6.1	0.074
Triglycerides (mg/dl)		135.7	± 23.1	133.8	± 21.7	135.7	± 23.7	137.3	± 23.8	0.582
LVEDD, mm		45.2	± 3.1	45.1	± 3.2	45.3	± 3.3	45.1	± 3.1	0.429
IVST, mm		9.8	± 1.1	9.7	± 1.0	9.8	± 1.1	9.8	± 1.1	0.613
LA diameter, mm		35.8	± 3.8	35.7	± 3.6	35.8	± 3.8	35.8	± 3.8	0.374

SBP: systolic blood pressure; DBP: diastolic blood pressure; LVEF: left ventricular ejection fraction; WBC: White blood cell count; LDL: low-density lipoprotein; HDL: high-density lipoprotein; LVEDD: left ventricle end-diastolic diameter; IVST: interventricular septum thickness; LA: left atrium.

* One-way ANOVA was performed to study differences among the three groups.

**Table 2:** Multinomial logistic regression analysis shows fragmented QRS is a predictor of non dipping in prehypertensive patients

Blood Pressure^a^	Variable	p	Odds Ratio	95% Confidence Interval
Dipper Prehypertension	Fragmented QRS	0.279	1.952	0.582-6.547
Non-dipper Prehypertension	Fragmented QRS	0.017	4.071	1.281-12.936

a The reference category is: Control.

## Discussion

The main finding of our study was that the frequency of fQRS was significantly higher in patients with non-dipper prehypertension compared to normotensives. Furthermore, the presence of fQRS on ECG was found to be a predictor of non-dipping in prehypertensive patients. To our knowledge, this is the first study to report the importance of fQRS in prehypertensive patients.

Prehypertension confers a high risk of progression to hypertension, and it may be associated with increased adverse cardiovascular events, inflammation, and target organ damage.^2^^,^^13^^,^^14^ Similarly to hypertension, prehypertension consists of non-homogeneous patients. Therefore, early identification of high-risk prehypertensives could lead to adequate prevention. Previous studies reported that deteriorated circadian blood pressure variability in prehypertensive patients may be associated with repolarization abnormalities detected by ECG.^15^ However, as a marker of depolarization abnormality, the importance of fQRS in prehypertensive patients is not clear. fQRS is a sign of inhomogenous ventricular conduction caused by myocardial scar, ischemia, or fibrosis.^8^ It has been shown that fQRS is a predictor of mortality and adverse cardiovascular outcomes in various cardiovascular diseases.^6^^-^^8^ Additionally, fQRS is a well described fibrotic factor in hypertension.^11^^,^^16^ It has been demonstrated that the frequency of fQRS is significantly higher in hypertensive patients than in normotensives,^9^ and non-dipper hypertensive patients have higher frequency of fQRS on ECG compared to hypertensive dippers.^10^^,^^11^ These studies revealed that increased blood pressure levels and elevated nighttime blood pressure levels are associated with the presence of fQRS on ECG in hypertensive patients, which indicates the higher fibrotic burden within myocardium in these patients.

Our study demonstrated that non-dipping blood pressure patterns are significantly associated with the presence of fQRS on ECG in prehypertensive patients, similarly to in hypertensive patients. Since the presence of fQRS on ECG is an important predictor of fibrosis and fibrotic burden within myocardium, the results of our study indicate a higher fibrotic burden in prehypertensive non-dippers compared to normotensives. The possible underlying mechanism for the association between fQRS and non-dipper blood pressure pattern in prehypertensives might be similar in hypertensive patients. Autonomic dysfunction-related increased sympathetic activity during nighttime, and chronic-continuous pressure overload related collagen fibers and connective tissue matrix accumulation within the myocardium might play the key roles for higher fibrotic burden and fibrosis in these patients.^17^^-^^19^

Non-dipping hypertension is a prognostic factor, and increased nighttime blood pressure levels indicate worse adverse cardiovascular outcomes compared to dipper patterns.^4^^,^^20^ Hence, definition of non-dippers is clinically important. In addition to being the precursor of hypertension, prehypertension includes a variety of patients who are at high risk for adverse cardiovascular events. Therefore, our results suggest that fQRS may be useful in defining the deteriorated circadian blood pressure variability which indicates high-risk prehypertensives.

Another aspect of our study is the importance of using 24-hour ABPM for detailed evaluation of blood pressure and final blood pressure pattern diagnosis. It is known that blood pressure patterns vary between ABPM and office records.^4^^,^^21^ Similarly, our study revealed that an important proportion of prehypertensive patients were not prehypertensive after 24-hour ABPM results. Since 24-hour ABPM is the gold standard for evaluation and diagnosis of hypertension, our study includes real prehypertensives.

Our study has some limitations. First, the study sample size is relatively small; however, the detection of prehypertensive patients is not an easy procedure in clinical practice. Second, our study included only newly diagnosed prehypertensive patients. Third, definition of prehypertension based on ABPM records is not clear. Hence, we designated patients with non-hypertensive elevated blood pressure as prehypertensives. Finally, lack of data regarding confirmation of fibrosis within myocardium by magnetic resonance imaging is another limitation.

## Conclusions

Fibrosis within myocardium is an important predictor of adverse cardiovascular events in patients with elevated blood pressure. fQRS is a simple and easily detectable ECG finding that indicates fibrosis within myocardium. This study revealed an important relationship between fQRS and non-dipper status in prehypertensive patients. We found that non-dipper prehypertensives have significantly higher frequency of fQRS compared to normotensives, and fQRS is an independent predictor of non-dipping in prehypertension. Our results suggest that fQRS may be useful in identifying high-risk prehypertensive patients before the development of hypertension. This identification may be helpful in terms of adequate prevention for future cardiovascular events. Future studies are necessary to demonstrate the prognostic value of fQRS in prehypertensive patients and to understand whether a more aggressive prehypertension treatment could normalize the ECG findings.
